# Predicting Ebola Severity: A Clinical Prioritization Score for Ebola Virus Disease

**DOI:** 10.1371/journal.pntd.0005265

**Published:** 2017-02-02

**Authors:** Mary-Anne Hartley, Alyssa Young, Anh-Minh Tran, Harry Henry Okoni-Williams, Mohamed Suma, Brooke Mancuso, Ahmed Al-Dikhari, Mohamed Faouzi

**Affiliations:** 1 GOAL Global, Dublin, Ireland; 2 University of Lausanne, Lausanne, Switzerland; 3 Institute of Social and Preventive Medicine, Lausanne, Switzerland; University of Oxford, UNITED KINGDOM

## Abstract

**Background:**

Despite the notoriety of Ebola virus disease (EVD) as one of the world’s most deadly infections, EVD has a wide range of outcomes, where asymptomatic infection may be almost as common as fatality. With increasingly sensitive EVD diagnosis, there is a need for more accurate prognostic tools that objectively stratify clinical severity to better allocate limited resources and identify those most in need of intensive treatment.

**Methods/Principal Findings:**

This retrospective cohort study analyses the clinical characteristics of 158 EVD(+) patients at the GOAL-Mathaska Ebola Treatment Centre, Sierra Leone. The prognostic potential of each characteristic was assessed and incorporated into a statistically weighted disease score. The mortality rate among EVD(+) patients was 60.8% and highest in those aged <5 or >25 years (p<0.05). Death was significantly associated with malaria co-infection (OR = 2.5, p = 0.01). However, this observation was abrogated after adjustment to Ebola viral load (p = 0.1), potentially indicating a pathologic synergy between the infections. Similarly, referral-time interacted with viral load, and adjustment revealed referral-time as a significant determinant of mortality, thus quantifying the benefits of early reporting as a 12% mortality risk reduction per day (p = 0.012). Disorientation was the strongest unadjusted predictor of death (OR = 13.1, p = 0.014) followed by hiccups, diarrhoea, conjunctivitis, dyspnoea and myalgia. Including these characteristics in multivariate prognostic scores, we obtained a 91% and 97% ability to discriminate death at or after triage respectively (area under ROC curve).

**Conclusions/Significance:**

This study proposes highly predictive and easy-to-use prognostic tools, which stratify the risk of EVD mortality at or after EVD triage.

## Author Summary

The unprecedented spread of EVD across the fragile healthcare systems of West Africa during the 2013–2015 outbreak infected over 28,600 patients and established it as a disease for which low-income countries are at disproportionate risk. In order to improve the standard of patient care, it is essential to better allocate scarce resources amongst the heterogeneous symptomatic presentations of EVD. This retrospective cohort study on 158 EVD(+) patients in Sierra Leone constructs 2 easy-to-use scoring systems that accurately stratify EVD severity and thus objectively identify the patients in most need of intensive therapy. Using statistically weighted symptoms and demographic characteristics, we obtained scores with a 91% and 97% ability to discriminate death at or after triage respectively. These scores included Ebola viral load, patient age and referral time as well as the symptoms of disorientation, haemorrhage and myalgia. Further univariate analysis revealed several independent predictors of mortality, where patients aged between 5 and 25 years were most likely to survive, while malaria co-infection increased the risk of death by 2.5-fold (p = 0.01). Correcting referral-time for viral load, we also quantify the benefits of early reporting as a 12% mortality risk reduction per day (p = 0.012). Mortality in this cohort was 3-fold more than patients treated in resource-rich settings (60.8% vs. 18%) and we propose that focused patient care is a feasible and low-cost effort that may begin to close this gap.

## Introduction

Ebola virus disease (EVD) caused by the virulent *Zaire ebolavirus* strain is described by the WHO as one of the world’s most deadly infections, with case fatality rates exceeding 80% in past epidemics [1, 2]. Supportive care in the 2013–2015 outbreak in West Africa was shown to reduce the EVD mortality rate to around 50% [3], and overall, the WHO has reported 40% fatalities among the 28,603 people affected by EVD [4]. Despite its notoriety as a fatal disease, over 80% of patients survived when treated in resource-rich environments of Europe and the USA [5]. Further, asymptomatic infections are not only possible but could constitute up to a third of all transmissions [6–9]. Overlooking these infected (but minimally contagious) individuals was proposed to result in the overestimation of EVD epidemic modelling, and revealed the heterogeneous range of EVD symptomology [10]. Improved prognostic tools that objectively stratify mortality risk among EVD patients could better allocate limited resources by identifying those most in need of intensive treatment and to aid clinical decision-making. Further, the clinical trials undertaken in the Ebola response have been criticised for the potential bias introduced via the lack of randomisation and contemporaneous controls [11, 12]; thus, a method of objectively controlling differences in mortality risk among participants may aid analysis.

Existing EVD staging models used in Sierra Leone, were based on a WHO protocol adapted from the clinical presentation of Lassa fever [13], where 3 symptomatic stages were described: 1) Early/non-specific, 2) gastrointestinal and 3) late/complicated, featuring haemorrhage and organ failure. While it has since been shown that these three stages of the disease are broadly correlated to EVD outcome [14], the system could be greatly improved by using statistically weighted symptoms that better stratify the risk of mortality. Several studies have already identified single symptoms statistically predictive for EVD mortality, such as confusion [15–17], diarrhoea,[16, 18] asthenia [15, 18], hiccups [14], haemorrhagic signs [14, 16, 19], dizziness [18], extreme fatigue [15], and high viral load [14, 17, 18, 20]. However, the various permutations in which symptoms occur in each individual, necessitates a multivariate approach to more accurately predict mortality.

The Ebola virus has been hypothesized to exercise its diverse range of virulence through the mammalian immune system. Here, it causes a pathologic overstimulation of innate immune receptors, triggering a flood of inflammation that causes collateral damage to multiple organ systems [21, 22], and results in a wide range of symptomatic presentations [8, 23]. It is then easy to imagine the additive detrimental effect of an inflammatory co-infection such as the malaria parasite, *Plasmodium falciparum*. The annual incidence of malaria in Sierra Leone is 350 cases per 1000 population and it has been reported to be more prevalent in EVD triaged patients than EVD itself [24]. However, despite these statistics, little is known about EVD/malaria co-infection or its effect on patient prognosis.

In this retrospective cohort study, we analyse the clinical and epidemiological data from 158 EVD(+) patients admitted to the GOAL-Mathaska ETC in Port Loko, Sierra Leone. We investigate the role of malaria in EVD pathogenesis and evaluate the potential of the clinical characteristics in predicting EVD mortality at triage as well as in on each day of patient care. Further, we use these results to construct a statistically weighted disease scoring and staging system, which identifies the most prevalent factors that are predictive of mortality.

## Methods

### Study design

This retrospective cohort study uses anonymized patient data collected between December 14, 2014 and November 15, 2015 at the GOAL ETC in Port Loko, Sierra Leone. Data comprised patient demographics, geographic location, clinical signs and symptoms, and laboratory results (for malaria infection and semi-quantitative Ebola viremia), as well as the final patient outcome of death or survival. We evaluate the potential of clinical characteristics in predicting EVD mortality and use these results to construct a symptom-based disease staging system, which corresponds to the prognostic power of the most prevalent symptoms.

### Patient referral

The ETC was run by the humanitarian organization GOAL Global in cooperation with the Sierra Leonean Ministry of Health and Sanitation (MoHS). The ETC opened in December 2014 and accepted 600 patients from a catchment area spanning 200km. EVD surveillance in Sierra Leone was implemented through District Ebola Response Centres (DERCs). Individuals who were sick were encouraged to report their illness (or the suspected illness of others) via the national or district Ebola call-lines. Individuals that met the WHO case definition for EVD [25], as well as those with confirmed EVD infection, were referred to the ETC from surrounding communities, holding centres, health facilities, and quarantine houses. All EVD(+) patients were treated according to standard treatment protocols developed by WHO and Médecins Sans Frontières [13, 26]. This included empiric antimalarial treatment (Artesunate and Amodiaquine), broad-spectrum antibiotics, and nutritional supplementation for all patients, as well as oral or intravenous fluid rehydration.

### Data collection

Signs and symptoms were recorded daily, on admission and throughout the patients’ length of stay at the ETC. Once triaged, blood was drawn and tested for EVD in on-site laboratories managed by Public Heath England. EVD diagnosis was determined by semi-quantitative reverse transcriptase-PCR (qRT-PCR) as previously described [27]. Briefly, the cycle threshold (Ct) value was used as an inverse proxy for viral load and a cut-off of 40 was used to discriminate between positive and negative values. Patients qualified as EVD(-) and were discharged from the ETC after returning two negative Ebola-specific qRT-PCR tests. Histidine-rich protein-II (HRP-II) antigen rapid diagnostic kits were used for testing of malaria infections, which were performed on admission at the ETC.

### Signs and symptoms

Symptoms were reported by the patient during a comprehensive questionnaire by trained staff. Haemorrhaging, pyrexia, and disorientation were recorded by clinicians after examination. Haemorrhagic signs included visible blood loss such as hematochezia, hematemesis, haematuria, epistaxis, haemoptysis or persistent haemorrhage from an IV catheter site as well as subcutaneous haemorrhage such as purpura and petechiae. Pyrexia was defined as a body temperature over 38°C, measured using an infrared thermal sensor. Disorientation was measured by trained ETC clinicians as per the AVPU alertness scale (where pain and unconsciousness were considered “disorientated”). Additionally, any specific mention of “confusion” or “disorientation” in the medical notes was also considered a positive for this variable.

### Cohorts and inclusion criteria

Of the 600 patients admitted to the ETC, 10 were declared dead on arrival and 24 were classified as late transfers from other ETCs or holding centres (treated elsewhere and thus convalescent on arrival) or had incomplete data. Thus, a total of 34 patients were excluded from this analysis. Of the 566 patients involved in the study, 100% had diagnostic test results for EVD, where 27.9% tested EVD(+) (n = 158). 543/566 patients had malaria test results, of which 34.6% were malaria(+) (n = 188). The cohort was evaluated for missing values in each variable. Referral time (the time in days from symptom onset to admission at the ETC) had 20 cases of missing data. Further analysis was undertaken to evaluate the aetiology of missingness, which included demographic variables (such as age and sex), clinical severity variables (such as EVD viral load) as well as the covariates used in the final scoring model. Here, we found that subjects with missing data did not differ systematically from those with observed referral time, which is in favour of the hypothesis that the data were missing completely at random. In addition, we performed a sensitivity analysis using the “Hotdeck” imputation technique, which showed that the model coefficients did not change when using complete data [28]. The patient catchment area and mortality rates can be visualised in **S1 Fig**.

### Data entry

Ethical approval for this research was granted by the Sierra Leone Ethics and Scientific Review Committee (SLESRC). To maximize data fidelity, patient files were entered into a secure Microsoft Excel database and crosschecked by 3 independent and trained analysts. Entry of clinical data was overseen by members of the clinical ETC staff. Graphs were constructed using Graphpad Prism, version 6. Univariate and multivariate analysis was conducted using STATA software, version 14 (StataCorp). Score validation was performed using “RMS” R-Package (R Development Core Team. ISBN 3-900051-07-0, URL: http://www.R-project.org). Results were deemed statistically significant at a p-value of less than 0.05.

### Primary data Analysis

Epidemiological data and symptoms were summarized by their frequencies and percentages. Univariate logistic regression was performed to assess the association between each predictor and the outcome of death (reported as Odds-Ratios (OR) and p-values). Potential interactions were tested (such as the effect of sex, age, referral time and Ebola contact). The functional relation between the outcome of death and continuous variables (age, days admitted, referral time and EVD viral load) were checked using a fractional polynomial model. The linearity assumption was confirmed for days admitted and referral time but not for age **(S2 Fig)** or Ct Value **(S3 Fig)**. To simplify the prognostic score, age was coded into three categories: (1) <5 years + [25–45] years, (2) [5–25] years, and (3) >45 years. **S2 Fig** shows the rationale for the chosen categories on their polynomial curve. The 5–25 group is used as a reference, being the lowest risk group. Comparing the <5 years category to the reference, we obtained an OR of 5.35 (p = 0.006), while the 25–45 category returned an OR of 2.61 (p = 0.002). Comparisons between the 0–5 and 25–45 groups, however, showed that they were not significantly different (p = 0.2) and they could thus be grouped to simplify the user interface of the score. The area under the ROC curves for the scoring systems presented in this study were not statistically different when comparing these age categorisations with the polynomial function of age as a continuous variable. However, a continuous function would undoubtedly be more accurate on a larger sample size.

For PCR results, a Ct value lower than 20 cycles was categorized as “high viral load” and correlated to the natural threshold for the probability of fatal outcome **(S3 Fig, dotted line)**. As there was an insufficient number of patients in the survival group (death = 96, survival = 62) compared to the number of 31 potential predictors, only the predictors associated to the outcome at level of p<0.20 were considered into a Stepwise Backward selection procedure to fit a multivariable logistic regression model [29]. The “daily” score for calculating risk after triage would ideally be handled with a time-dependent model in order to limit immortal time bias. However, these were not a good fit for our data, as the proportionality-hazards assumption was violated by non-parallel lines between categorical variables on log-log plots. Thus, a logistic multivariate model was privileged. Model diagnostics were performed to check for influential observations that impact coefficient estimates and a Hosmer-Lemeshow goodness-of-fit test was performed to assess calibration. Discriminative performance of the final model was assessed by calculating the Area Under the Receiver Operating Characteristics (ROC) Curve (AUC) and its 95% confidence interval.

### Calculation of the prognostic scores and model validation

The β-coefficient = log(OR) of each covariate of the final model was converted into an integer-based point-scoring system. The score was then derived as the sum of the covariates’ weighted scores. Internal validation using the bootstrap method (repeated 1000 times) as described in Harrell et al [30] was used to provide a more accurate estimate of the performance of the original model (model based score: AUC_original_). The algorithm calculates the optimism of the predictive discrimination in the original model. The difference (AUCoriginal−optimism) gives the bootstrap-corrected (i.e. internally validated) performance of the original model. As described in Steyerberg et al [31], bootstrapping has unavoidable limitations in small cohorts with a large number of predictors.

### Role of the funding source

The funder had no role in study design, data collection, data analysis, data interpretation, or writing of the report. The corresponding author had full access to the data in the study and had final responsibility for the decision to submit for publication.

## Results

### 1. Epidemiological characteristics of EVD outcome

**Fig 1:**
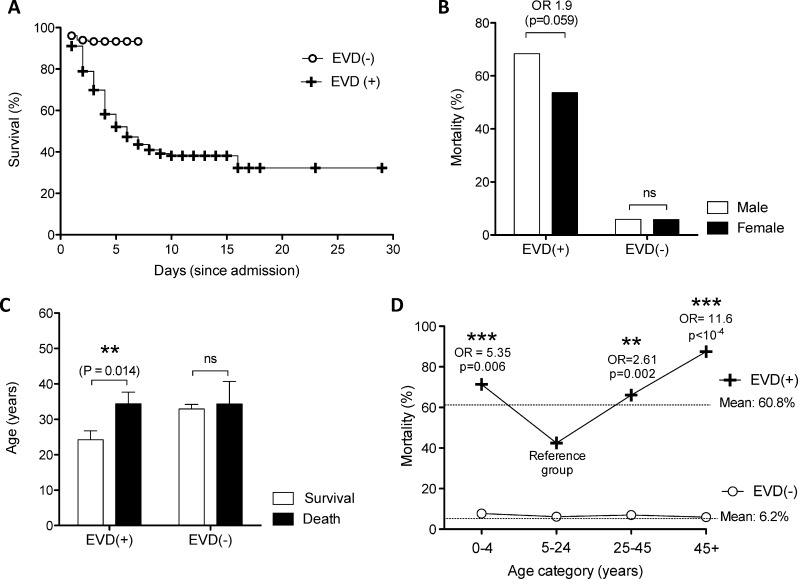
Epidemiological characteristics of EVD outcome. **(A)** Kaplan-Meier survival analysis of patients in the ETC according to their EVD status. **(B)** Mortality among EVD(-) and EVD(+) admissions according to gender. **(C)** Average age of death among EVD(-) and EVD(+) patients. **(D)** Mortality rate across age groups in EVD(-) and EVD(+) cohorts. Dotted lines represent the average mortality rate across all ages in the cohort. Statistics in **(C)** calculated by unpaired t test *: p<0.05, **: p<0.005, ***: p<0.001, ns: not significant.

Of the 566 patients included in this study, 27.9% tested positive for EVD (n = 158). The crude mortality rate among EVD(+) patients was 60.8% **(Fig 1A)**. Mortality rates were slighter higher in males (68.4% vs. 53.7%), with a statistically insignificant 1.9 fold increase in odds of death (p = 0.06) **(Fig 1B)**. EVD survivors were on average 10 years younger than those who died (24.9 years vs. 34.3 years, p = 0.014) **(Fig 1C)**. However, mean age of death did not differ among EVD(-) patients who died before being transferred out of the ETC **(Fig 1C)** or among genders (p>0.05). In general, case fatality rates for EVD were higher at the youngest and oldest extremes of age. The patient group aged between 5 and 24 years had the lowest mortality rate of 42.5%, which was significantly lower than other age groups. The over-45’s and under-5’s were particularly vulnerable, being 11.6 and 5.4 fold more likely to die, respectively **(Fig 1D)**. Age groups were selected in order to ensure the mathematic simplicity of the final score. The polynomial curve of this continuous variable is shown in **S2 Fig**. Categorisation did not significantly alter the accuracy of the final scores (p>0.05).

### 2. Prognostic potential of clinical characteristics recorded at and after admission

**Fig 2:**
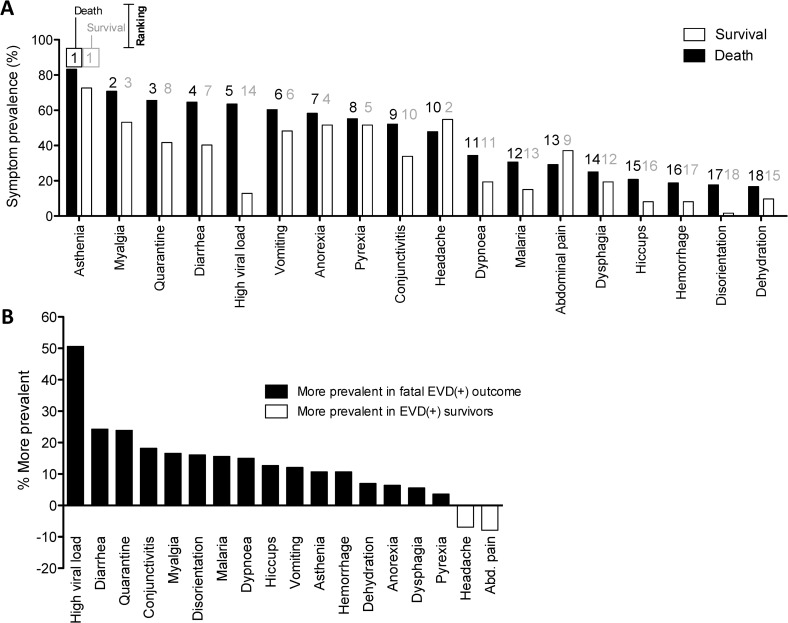
Prognostic potential of clinical signs and symptoms recorded at and after admission. **(A)** Prevalence of clinical characteristics at triage amongst EVD(+) patients who either survived or died, ranked according to the prevalence in fatal outcomes. Rankings from 1–19 are listed above each bar: black for the outcome of death and grey for survival. **(B)** Differences in symptom prevalence between EVD survivors and those who died. Positive values are more prevalent in fatal outcomes. Negative values are more prevalent in survivors.

In an effort to better predict the risk of EVD death, we analysed the prevalence and prognostic potential of the major clinical characteristics among EVD(+) patients. Triage symptoms reported by over 50% of fatal EVD(+) patients were asthenia, myalgia, diarrhoea, anorexia, vomiting, pyrexia, and headache **(Fig 2A and Table 1)**. The prevalence of several of triage symptoms was notably different between fatal and non-fatal outcomes, as can be seen by comparing their ranking **(Fig 2A)** or their differential prevalence **(Fig 2B)**. As expected, high viral load (Ct value <20) was approximately 50% more common among fatal outcomes and univariate analysis revealed it as a major correlate of mortality with 11.8 fold odds of death (p>0.0001) **(Table 1)**. While disorientation on admission was not common in EVD(+) patients (11.4%), when present, it was associated with 94.4% of fatalities in EVD(+) patients, and was therefore the strongest indicator of fatal outcome (OR 13.1, p = 0.014) **(Table 1)**. Other factors showing a statistically significant association with death were diarrhoea, hiccups, myalgia, dyspnoea and conjunctivitis (all p<0.05) **(Table 1)**. While haemorrhagic signs were infrequent on admission (14.6%), developing haemorrhage at any point during admission at the ETC was associated with a 6-fold higher odds of mortality (p>0.0001) **(Table 1)**. Finally, malaria infection was more prevalent in fatal outcomes **(Fig 2B)** and will be discussed further below.

**Table 1:** Association of clinical and laboratory characteristics to EVD mortality.

Clinical/Laboratory characteristics reported at triage	Prevalence				Univariate		Multivariate			
	Patients with characteristic who survived		Patients with characteristic who died		Unadjusted association of characteristic with mortality		Multivariate association of selected characteristics with mortality			
	%	*(n)*	%	*(n)*	OR	*P value*	OR	*P value*	Coeff.	Weight^‡^
**TOTAL**	39.2	*(62)*	60.7	*(96)*	-		-			
**Ct value (<20)†**	12.9	*(8)*	63.5	*(61)*	11.8	*0*.000*	0.9	*0*.*900*	-0.1	**+ 0**
**Diarrhoea**	40.3	*(25)*	64.6	*(62)*	2.7	*0*.003*				
**Conjunctivitis**	33.9	*(21)*	52.1	*(50)*	2.1	*0*.026*				
**Myalgia**	53.2	*(33)*	69.8	*(67)*	2.0	*0*.036*	3.7	*0*.02*	1.3	**+ 3**
**Disorientation**	1.6	*(1)*	17.7	*(17)*	13.1	*0*.014*	38.2	*0*.*074*	3.6	**+ 7**
**Malaria infection**	15.0	*(9)*	30.6	*(26)*	2.5	*0*.034*				
**Dyspnoea**	19.4	*(12)*	34.4	*(33)*	2.2	*0*.044*				
**Hiccups**	8.1	*(5)*	20.8	*(20)*	3.0	*0*.038*				
**Vomiting**	48.4	*(30)*	60.4	*(58)*	1.6	*0*.*138*				
**Asthenia**	72.6	*(45)*	83.3	*(80)*	1.9	*0*.*107*				
**Haemorrhage**	8.1	*(5)*	18.8	*(18)*	2.6	*0*.*070*				
**ORL haemorrhage**	3.2	*(2)*	10.4	*(10)*	3.5	*0*.*115*				
**Dehydration**	9.7	*(6)*	16.7	*(16)*	1.9	*0*.*221*				
**Anorexia**	51.6	*(32)*	58.3	*(56)*	1.3	*0*.*407*				
**Dysphagia**	19.4	*(12)*	25.0	*(24)*	1.4	*0*.*410*				
**Pyrexia**	51.6	*(32)*	55.2	*(53)*	1.2	*0*.*658*				
**Hepatomegaly**	1.0	*(1)*	4.2	*(4)*	2.7	*0*.*388*				
**Anuria**	1.6	*(1)*	4.2	*(4)*	2.7	*0*.*388*				
**Headache**	54.8	*(34)*	47.9	*(46)*	0.8	*0*.*396*				
**Abdominal pain**	37.1	*(23)*	29.2	*(28)*	0.7	*0*.*299*				
**Ref group**: **Age (5–25)**	40.3	*(25)*	14.6	*(14)*	*-*	*-*	*-*	*-*	-	**+ 0**
**Age (<5 and 25–45)**	53.2	*(33)*	58.3	*(56)*	3.3	*0*.000*	8.2	*0*.001*	2.1	**+ 4**
**Age (>45)**	6.5	*(4)*	27.1	*(26)*	11.6	*0*.006*	80.9	*0*.000*	4.4	**+ 9**
**Ct value (>20)**	87.1	*(54)*	36.5	*(35)*	0.70.9	*0*.000*				
**Referral time (mean)**	4.6	*days*	3.9	*days*		*0*.*120*	0.6	*0*.000*	-0.5	**- 1**
**Ct (<20) X referral time†**	-	*-*	-	*-*	1.4	*0*.008*	2.3	*0*.005*	0.8	**+ 2**
**Clinical/laboratory characteristics reported at any time AFTER triage in the ETC** *(only significant results listed)*										
**Disorientation**	14.5	*(9)*	66.7	*(64)*	11.8	*0*.000*	138.6	*0*.000*	4.9	**+ 10**
**Haemorrhage**	6.5	*(4)*	24.0	*(23)*	6.0	*0*.000*	17.2	*0*.001*	2.8	**+ 6**
**Age (<5 and >25)**	59.7	*(37)*	85.4	*(82)*	3.3	*0*.000*	8.1	*0*.012*	2.1	**+ 4**
**Ref group**: **Age (5–25)**	40.3	*(25)*	14.6	*(14)*	*-*	*-*	*-*	*-*	*-*	**+ 0**
**Days admitted (mean)**	10.4	*days*	4.2	*days*	0.69	*0*.000*	0.5	*0*.000*	-0.6	**+ 1**

Clinical and laboratory characteristics found at triage **(upper section)** or at any time during the patient’s stay at the ETC after triage **(lower section)**.

Characteristics appear in order of their differential prevalence (mortality—survival).

The “**Univariate**” column shows the unadjusted OR of each characteristic to mortality (shaded with a heat map identifying the most predictive characteristics).

The “**Multivariate**” column presents the characteristics used in the triage score (upper section) and daily score (lower section). Coefficients (**Coeff**) and their mathematically manipulated score weightings are shown in the final column.

^**‡**^ Score weights are calculated as 2 X coefficient, rounded off to the nearest whole integer.

† “**Ct (<20)**” appears twice: First as an unadjusted variable and later as “**Ct (<20) X Referral time**” where it represents an interaction with referral time to aid mathematic simplicity of the score.

*: p<0.05, OR: Odds ratio, Coeff: coefficient, ETC: Ebola treatment centre.

### 3. Prognostic value of Ebola virus load (Ct value)

**Fig 3:**
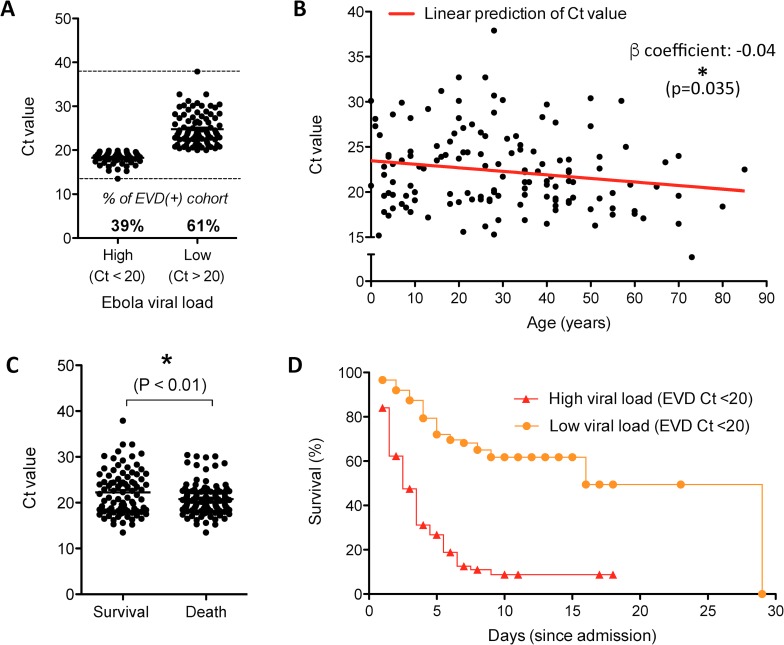
Prognostic value of Ebola virus load (Ct value). **(A)** Distribution of Ct values^1^ for EVD(+) patients considered to have a high viral load (Ct ≤ 20) and low viral load (Ct > 20). **(B)** Ct value distribution across age in the EVD(+) cohort. The red line plots the fractional polynomial prediction of the Ct value. **(C)** Ct values amongst survivors and fatalities in the EVD(+) cohort. **(D)** Kaplan-Meier survival analysis of EVD(+) patients according their Ebola virus loads, either considered as high viral (Ct ≤ 20) or low viral load (Ct > 20). ^1^ Ct values represent Ebola-specific qRT-PCR results (inversely proportional to the viral load). Statistics in **(C)** calculated by unpaired t test *: p<0.05, **: p<0.005, ***: p<0.001, ns: not significant.

EVD diagnoses were routinely confirmed by qRT-PCR, where the cycle threshold (Ct) value is inversely proportional to the Ebola virus copy number. We used similar parameters presented in other studies to delineate high and low Ct values [14, 32], where a Ct value lower than a threshold of 20 cycles was categorized as “high viral load”. This also correlated to the natural threshold for the probability of fatal outcome in our cohort **(S3 Fig)**. Ct values were available for 144/158 of the EVD(+) cohort (91.1%) and ranged from 13.5 to 37.9. Of these, 39% (n = 57) were classified as having with high viral loads **(Fig 3A)**. The mean Ct value (22.0) did not vary by gender (p>0.05) but was differentially distributed across ages, where each 10 years corresponded to a decrease of 0.4 Ct points (i.e. an increase of viral load) (p = 0.035) **(Fig 3B)**. Finally, the average Ct value for survivors was significantly higher than those who had a fatal outcome (24.9 vs. 20.6, p<0.01) **(Fig 3C and 3D)**, where odds of death were 12.6 times higher for patients with Ct values of less than or equal to 20 (p<0.0001).

### 4. Impact of EVD referral sensitivity and admission time on outcome

**Fig 4:**
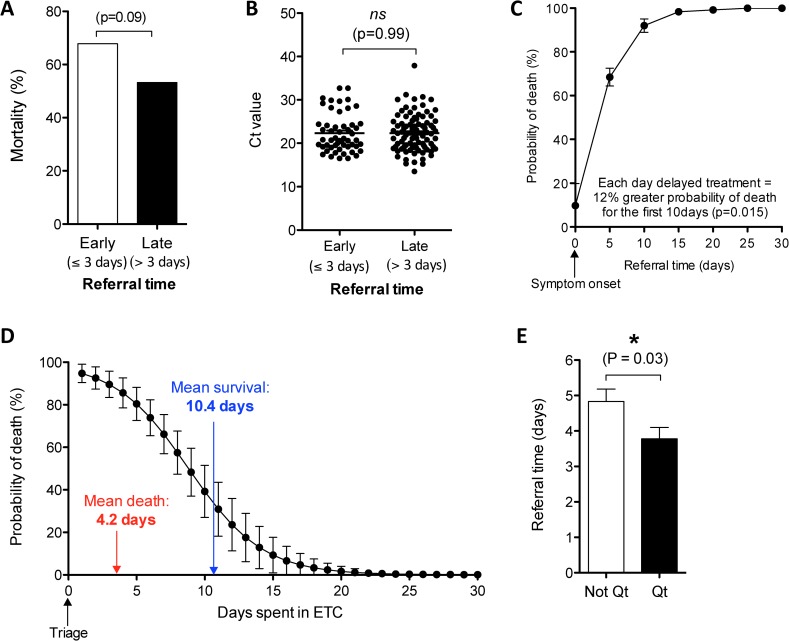
Impact of EVD referral sensitivity on diagnosis and patient outcome. **(A)** Mortality rate for EVD(+) patients who were referred “early” (within 3 days of reported symptom onset) or “late” (after 3 days of symptom onset). **(B)** Cycle threshold (Ct) values^1^ among early and late EVD(+) referrals. **(C)** Probability of death according the referral time corrected for Ct value. **(D)** Probability of death according the number of days spent in the ETC. The average number of days spent in the ETC for patients with fatal or survival outcomes are indicated in colour. **(E)** Mean referral time of EVD(+) patients according to quarantine (Qt) status. ^1^ Ct values represent Ebola-specific qRT-PCR results (inversely proportional to the viral load). Statistics in **(A, B and E)** calculated by unpaired t test *: p<0.05, **: p<0.005, ***: p<0.001, ns: not significant

An EVD staging model developed by the UK Defense Medical Services in reference to WHO guidelines on the pathogenesis of haemorrhagic fever, divides the temporal evolution of EVD into three symptomatic stages [13, 14]. Here, the “early” stage is comprised of non-specific symptoms lasting three days. Considering this timeline, we investigated the impact of early referral on mortality. Crude analysis showed that a fatal outcome was not associated to a later referral time. Oppositely, EVD survivors presented at the ETC one day later than those who died (4.6 vs. 3.6 days) albeit a statistically insignificant trend (p = 0.12). Indeed, those presenting within 3 days of symptom onset had a 15% higher mortality than those presenting later (p = 0.09) **(Fig 4A)**. This counter-intuitive trend of earlier healthcare seeking behaviour resulting in higher death risk could be theoretically explained as the confounding effect of disease severity. Here, we propose that patients presenting earlier are doing so as they have a more severe acute disease and thus represent a population predisposed to mortality risk. To investigate this hypothesis, we compared viral loads as a proxy for disease severity and found that those presenting earlier had similar viral loads to those presenting later **(Fig 4B)**. This result indicates that early presenters have more severe acute disease. Correcting for viral load as a confounding factor (where comparisons are only made between patients with equal viral loads), we found the more intuitive result that delayed treatment was significantly associated with mortality. Here, the probability of death increased by an average of 12% for each day of delayed treatment during the 1^st^ week of symptoms (p = 0.012) **(Fig 4C) (Table 1)**. This quantifies the benefits of early health care seeking behaviour.

Oppositely, each day spent within the ETC increased the odds of survival by 1.4 fold (p<0.0001) irrespective of viral load **(Fig 4D) (Table 1)**. The average admission duration for EVD(+) survivors was 10.4 days while deaths occurred, on average, within the first 4.2 days of admission **(Fig 4D)**.

Finally, quarantine status upon admission was available for 96% of ETC admissions (n = 551), 25% of which were referred from quarantine houses. EVD(+) patients referred from quarantined homes had an earlier referral time than those not referred from quarantine facilities (3.8 days vs. 4.7 days, p = 0.03) **(Fig 4E)**. This quantifies the potential patient benefit of quarantine in the region, however, we found no difference in mortality by quarantine status (OR = 1.8, p = 0.08).

### 5. Impact of malaria infection on patient outcome

**Fig 5:**
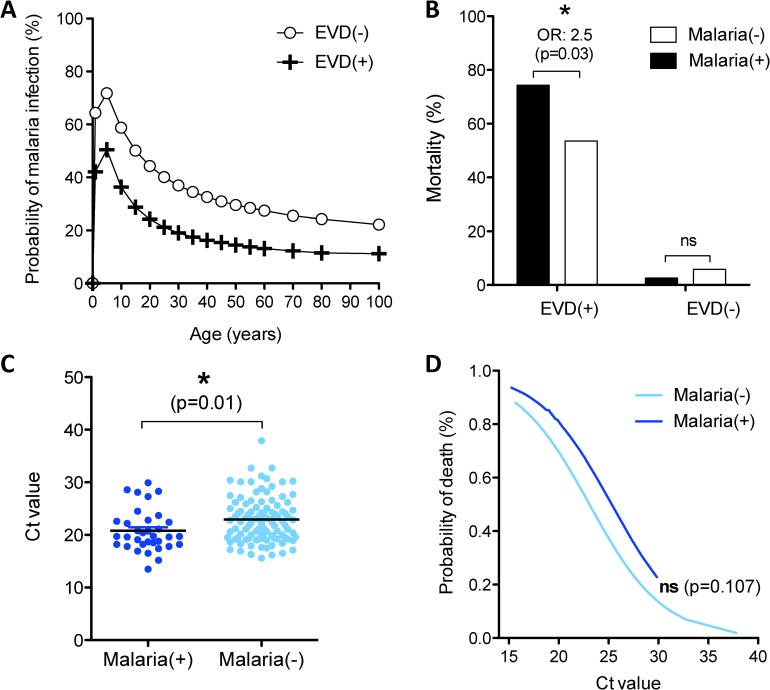
Impact of malaria co-infection on patient outcome. **(A)** Probability of malaria infection across age for EVD(-) and EVD(+) cohorts. **(B)** Mortality rates among EVD(-) and EVD(+) cohorts according to the presence of malaria infection. **(C)** Ct value^1^ among EVD(+) patients co-infected or not by malaria. **(D)** Probability of death among EVD(+) patients infected or not by malaria according to EVD Ct value^1^. ^1^ Ct values represent Ebola-specific qRT-PCR results (inversely proportional to the viral load). Statistics in **(C)** calculated by unpaired t test. *: p<0.05, **: p<0.005, ***: p<0.001, ns: not significant

Of the 543 EVD(+) and EVD(-) patients with a known malaria test, 34.8% tested malaria(+). Among EVD(+) patients, 24% were co-infected with malaria compared to 38% EVD(-) (OR = 2, p = 0.005). The prevalence for malaria infection varied drastically across age categories, where 5 year olds had an over 50% probability of being malaria(+) in both EVD(-) and EVD(+) cohorts **(Fig 5A)**. Despite the WHO standard of care to treat all ETC admissions with Arteminisin Combination Therapy (ACT) upon admission (irrespective of malaria status)[33], EVD(+)/malaria(+) co-infected patients suffered a significantly higher mortality rate compared to EVD alone (74.3% vs. 53.6%, OR = 3.9, p = 0.03 after controlling for age and gender) **(Fig 5B)**.

Ebola viral load was differentially distributed across EVD(+) patients who were co-infected or not with malaria. Co-infected EVD(+)/malaria(+) patients had significantly higher viral loads compared to patients infected with EVD alone (mean Ct = 20.8 vs. 22.3, p<0.01) **(Fig 5C)**. Controlling for viral load, the increased mortality in malaria co-infected EVD(+) patients was abrogated (p = 0.107) **(Fig 5D)**. Taken together, these results reveal a potential pathogenic synergy between the malaria parasite and Ebola virus.

### 6. Derivation of a prognostic scoring system for EVD outcome

Performing multivariate analysis of the above data, we selected the clinical characteristics most predictive for EVD mortality using data collected at triage or at any time during the patients’ stay in the ETC **(Table 1)**. By stepwise backwards elimination, and prioritizing the most prevalent symptoms, we identified several characteristics which yielded significant predictive values at triage and during admission. Characteristics that were statistically significant predictors of mortality at admission were vulnerable age groups (<5, 25–45 and >45 years), myalgia, disorientation and referral-time (normalised to viral load) **(Table 1)**. Characteristics that were statistically significant predictors of mortality after admission were vulnerable age groups (<5 and >25 years), disorientation and haemorrhage. Oppositely, days spent in the ETC was a significant predictor of survival (OR 1.5-fold for each day, p<0.0001) **(Table 1)**. Despite the strong association of malaria co-infection with fatality in our univariate analysis above, malaria infection was rendered insignificant in our multivariate analysis.

**Fig 6:**
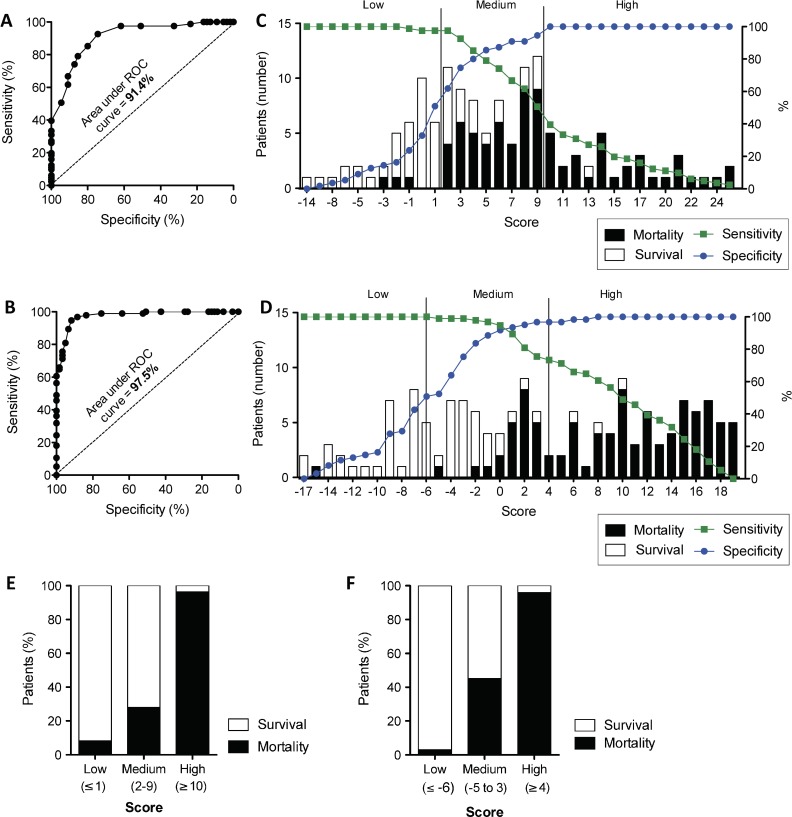
Derivation of prognostic scoring system for EVD outcome. The sensitivity and specificity of predicting mortality in EVD(+) patients using the scoring system developed with clinical parameters collected **(A)** at triage or **(B)** daily during the admission of the patient at the ETC. The area under the receiver-operator characteristic (ROC) curve represents the discriminative power of each score. **(C-D)** Sensitivity (green) and specificity (blue) according to the score points of **(C)** the triage score and **(D)** the daily score. Prevalence of survivors and those with fatal outcome are displayed as bar graphs and risk category cut-offs are shown as vertical lines. **(E)** Percentage of survivors and patients with fatal outcome classified in each risk category of the triage EVD mortality score and the **(F)** daily EVD mortality score.

**Fig 7:**
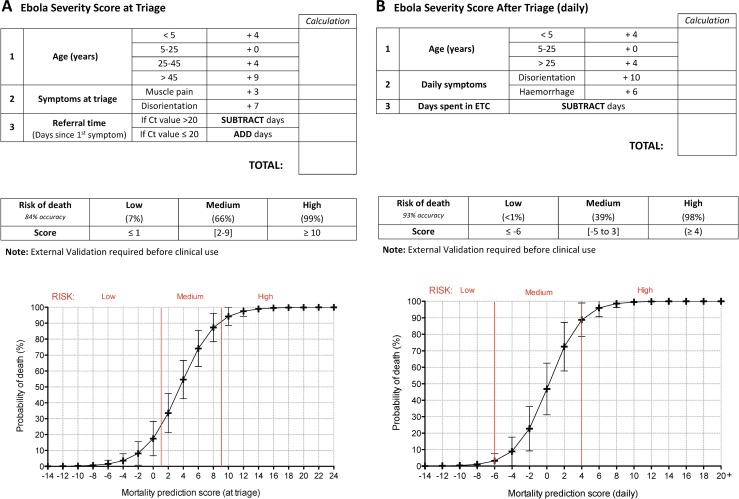
Scorecards to extrapolate the Ebola severity risk. Ebola Virus disease severity risk calculated at **(A)** triage and **(B)** after triage. Full page printable templates of these scorecards can be found in the supplementary information **(S4 and S5 Figs)**.

We then calculated weightings for both scores from the predictive coefficients with the aim to find a simplified scoring model using whole integers and calculations limited to subtraction or addition **(Table 1)**. Testing the sensitivity and specificity of these weightings for the prediction of EVD infection, we found that the characteristics yielded an area under the ROC curve (AUC) of 91.4% (CI95%: 87–96%) for discriminating mortality at triage **(Fig 6A)** and 97.5% (CI95%: 95–99%) for calculations after admission **(Fig 6B)**. The risk category cut-offs for each score are illustrated in **Fig 6C and 6D**. Each category contains at least 10% of the cohort. The 3 risk cut-offs (Low, Medium and High) were selected based on the linear risk curve **(Fig 7)**, where “Low” and “High” categories represent risk plateaus on the extremes of the risk statistic (Low <7%, and High >98%).

Examining the accuracy of the triage mortality score, we found that the “high” risk classification was composed of 99% correctly classified fatal outcomes while the “low” risk category had a less than 10% mortality rate **(Fig 6E)**. After triage, the “high” risk category of the daily score was composed of 98% fatalities compared to less than 1% in the “low” risk category **(Fig 6F)**. An internal validation of the triage and daily scores yielded a final discriminative power of 89.12% and 97.04% respectively **(Table 2)**. As described in Steyerberg et al [31], bootstrapping has unavoidable limitations in small cohorts with a large number of predictors and thus the optimism may be over-estimated. External validation is needed to best test these associations.

**Table 2:** Internal validation of EVD prognostic scores.

	AUC_original_	Optimism	AUC_corrected_
**Triage mortality score**	91.4%	0.022%	89.12%
**Daily mortality score**	97.56%	0.005%	97.04%

## Discussion

The Ebola virus affects diverse tissues types across various organ systems [8, 21–23] and it is thus unsurprising that infection outcomes have a similarly heterogeneous range: from asymptomatic to multiple organ failure. This heterogeneity presents a situation where blanket therapies would be not only inefficient, but also inappropriate. Improving the efficiency of interventions in the difficult working conditions of an ETC is a priority in resource constrained environments, where at some points of the epidemic, EVD patients could expect less than 20min of clinician care per day [34]. In an effort to create an objective measure of disease severity, we evaluated the clinical features of EVD, which are able to discriminate between death and survival.

As with all the studies reporting on symptomatic outcomes in the Ebola outbreak, analysis is limited by the accuracy of self-reported symptoms that are subject to recall-bias as well as the potential social stigma associated to withholding symptoms, leading to acquiescence or social desirability bias. However, the generalizability of the data collected on this cohort was demonstrated when we externally validated a diagnostic scoring system by Levine et al and obtained an almost identical discriminative power compared to their cohort from rural Liberia [35]. Further, many of our descriptive findings were consistent with other studies on EVD mortality, where the patients most likely to have a fatal outcome were those over 45 or under 5 years of age **(Fig 1D)** [14–16, 18], and where males and females had similar mortality rates **(Fig 1B)** [36]. Symptoms predictive of EVD related mortality in our cohort were disorientation, diarrhoea, hiccups, myalgia, dyspnoea, and conjunctivitis **(Fig 2A and 2B, Table 1),** which differ slightly from other studies, where disorientation [16, 18] and diarrhoea [15, 16, 18] were the only overlapping symptoms predictive of fatal outcome. In line with other studies, our results show that Ebola viral load is a significant determinant of patient outcome, where mortality was over 12 times more likely when patients had a Ct value ≤20 **(Table 1)** [3, 18, 32, 37].

A recent report on the heterogeneous kinetics of Ebola viral load in blood revealed stark differences between survivors and non-survivors [38], and helps to explain our counterintuitive finding that morality rates were slightly higher among patients with earlier referral times. Here, we show the existence of “survival-bias”, where those presenting earlier may have had more severe acute disease and thus a higher predisposition for mortality, while late-presenters tended to survive. Controlling for viral load, we can conclude the expected finding, that referral time was a significant determinant of mortality **(Fig 4C)**, where each day without treatment augmented mortality risk by over 12% within the first week of symptoms. This information supports the important public health message on the benefits of early referral and the efficacy of supportive care.

In this cohort, malaria co-infection was a prevalent and significant determinant of EVD mortality where co-infected patients had a 2.5-fold increased odds of death **(Fig 5B)**. As malaria prevalence was most common among children in this cohort, it is likely to have played a role in their increased mortality. A study on a smaller cohort of 89 patients in Guinea did not find malaria as a significant determinant of mortality, albeit limited by a number of 24 control patients [39]. Another recent study revealed that patients treated with artesunate-amodiaquine therapy had a 31% lower risk of death compared to artemether-lumefantrine [40]. Fortunately, all patients in this study received the antimalarial associated with survival. Our analysis showed that the correlation of mortality to malaria co-infection was confounded by Ebola viral load, where malaria co-infection was significantly associated to increased viremia **(Fig 5C)**. The fact that adjusting malaria co-infection for Ebola viremia abrogated malaria’s association with mortality reveals a potential synergy between the malaria parasite and the Ebola virus, where malaria may increase mortality via increased EVD virulence. As malaria parasitemia was not quantified in this study, it is unknown whether the effect of malaria infection had a linear association with EVD, or indeed, whether it was reciprocal. While the mechanism of this potential interaction is unknown, the virulent synergy between viruses and parasites has been previously described to act via the immune system and deserves further investigation [41, 42].

After analysing the potential interactions, we included the most prevalent and predictive clinical characteristics of EVD into two prognostic scores, stratifying the risk of mortality at triage and in daily clinical care. We obtained a discriminative power of over 90% for both scores and, using our cohort, we were able to predict high-risk outcome of death with over 95% accuracy **(Fig 6E and 6F)**. It is well appreciated that the accurate diagnostic triage of EVD is essential to reduce the risk of nosocomial infection. However, prognostic triage may play an equally important role in patient safety by focusing intensive care on those who need it most.

### Limitations

All prognostic tools carry the risk of becoming self-fulfilling prophecies if incorrectly used as an indicator for palliation: dooming severely ill patients to death when the score is not reflective of clinical advances. This score is specifically adapted to an Ebola response in resource-constrained settings, where clinical resources achieved a 40% survival rate. As an 80% survival rate was possible among patients in resource rich environments [5], it is clear that the interpretation of the score would need to evolve with anticipated clinical advancements. However, the major asset of this score is not limited to prediction of the binary outcome of death, but rather its use as a proxy for “disease severity” in resource limited environments. Thus, while the outcome of “death” may change with improved treatment options, patients scoring highly on this tool can still be shortlisted for intensive intervention. Additionally, with the exciting potential of machine-learning predictive tools [43], scoring systems such as these can become more durable and evolve with their developing environments, where a future of accurate EVD diagnosis and prognosis is a realistic possibility.

As we found for malaria, EVD is certainly not the only contributing factor to mortality within an ETC, and patients who have lived a lifetime within a poorly resourced health care system very probably have diverse and complex competing risks. This is an unavoidable bias, as accurate secondary diagnostics for co-morbidities were primitive at best for the bulk of the patients. We await retrospective analyses on patient samples that may reveal the presence of other co-infections or confounding genetic/immunologic anomalies.

Finally, external validation is an essential step before the endorsement of any clinical tool. Without external validation, the level of inaccuracy within this cohort cannot be estimated and thus these scoring systems must be used with this caution in mind.

### Conclusion

This study identifies several epidemiological and clinical features, which are significantly predictive for the outcome of EVD infection and proposes several highly accurate statistical tools to predict the clinical severity of EVD and aid objective clinical prioritization.

### Perspectives

External validation and systematic meta-analyses of the clinical features of EVD are needed to fine-tune the statistical weightings of this score to further improve its accuracy and geographical relevance.

## Supporting Information

S1 Checklist STROBE Checklist. (DOCX)

S1 Fig EVD(+) mortality rate by section at the GOAL-Mathaska ETC. (TIF)

S2 Fig Probability of death across ages. Age categories were made to ensure mathematic simplicity of the clinical scores developed in this study. Dotted lines show the age categorisations based on the risk of death. As the lowest risk group, the 5–25 year olds are used as a reference. Comparing the 0–5 year olds to the reference, we obtained an OR of 5.35 (p = 0.006), while the 25–45 category returned an OR of 2.61 (p = 0.002). Comparisons between the 0–5 and 25–45 groups, however, showed that they were not significantly different (p = 0.02). This result qualifies the 0–5 and 25–45 age groups for pooling. The area under the ROC curves for the scoring systems presented in this study were not statistically different when comparing these age categorisations with the polynomial function of age as a continuous variable.
(TIFF)

S3 Fig Probability of death according to Ct value. **(A)** Predicted risk of death using fractional polynomial analysis. **(B)** Predicted risk of death (%). The dotted vertical lines indicate the threshold for “high viral load” (Ct<20) and “low viral load” (Ct>20).
(TIFF)

S4 Fig Printable scorecard to extrapolate the Ebola severity risk at triage. (TIFF)

S5 Fig Printable scorecard to extrapolate the Ebola severity risk after triage. (TIFF)

S1 Table Complete data for the multivariate score to predict Ebola severity risk at triage. (DOCX)

S2 Table Complete data for the multivariate score to predict Ebola severity risk after triage. (DOCX)
