# Supplementary figures and images for: Predicting Ebola Severity: A Clinical Prioritization Score for Ebola Virus Disease
Source: PLoS Negl Trop Dis. 2017 Feb 2;11(2):e0005265. doi: 10.1371/journal.pntd.0005265 (PMC5289426; doi:10.1371/journal.pntd.0005265)

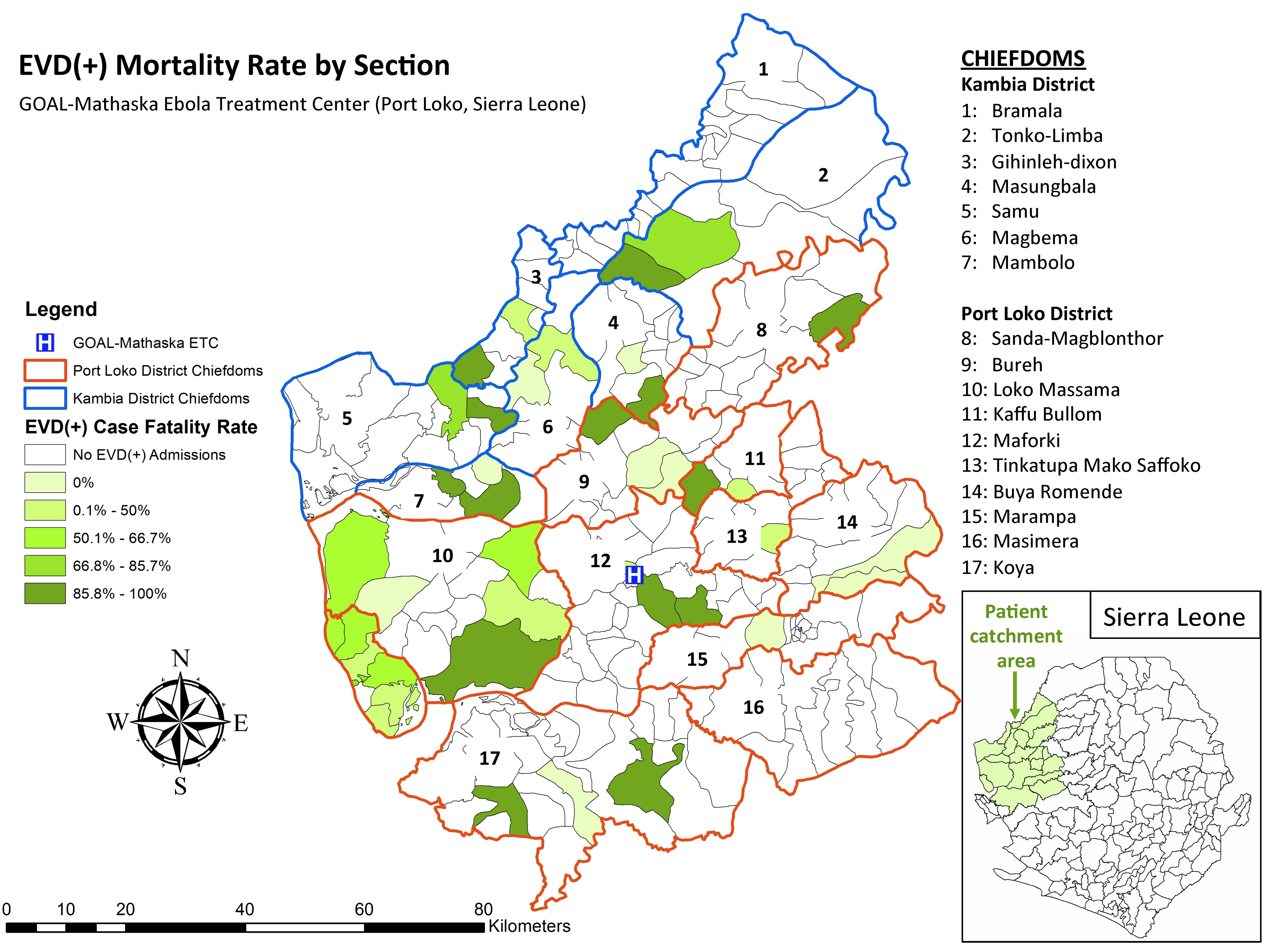

Supplement: S1 Fig — (TIF) [file pntd.0005265.s002.tif]

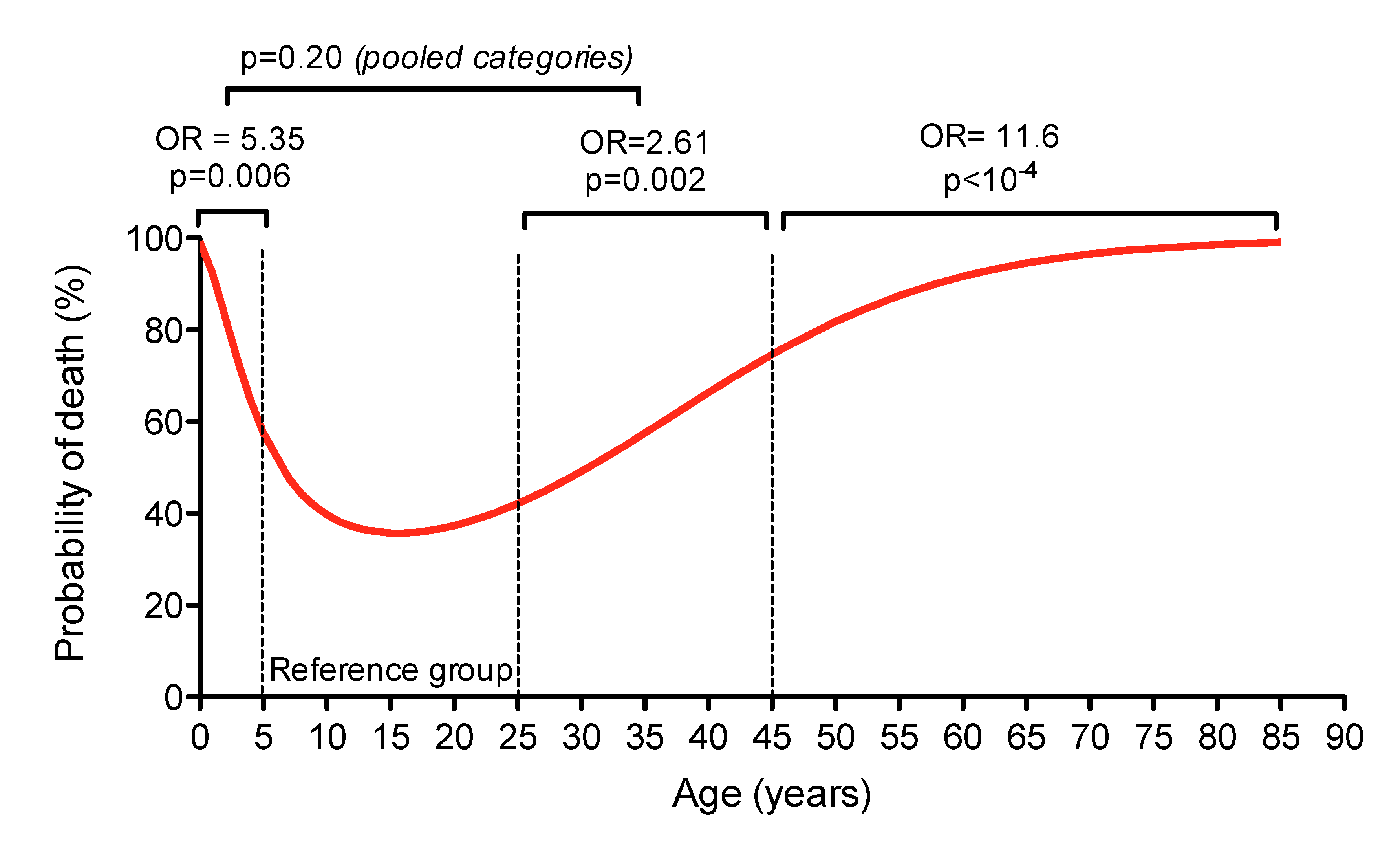

Supplement: S2 Fig — Age categories were made to ensure mathematic simplicity of the clinical scores developed in this study. Dotted lines show the age categorisations based on the risk of death. As the lowest risk group, the 5–25 year olds are used as a reference. Comparing the 0–5 year olds to the reference, we obtained an OR of 5.35 (p = 0.006), while the 25–45 category returned an OR of 2.61 (p = 0.002). Comparisons between the 0–5 and 25–45 groups, however, showed that they were not significantly different (p = 0.02). This result qualifies the 0–5 and 25–45 age groups for pooling. The area under the ROC curves for the scoring systems presented in this study were not statistically different when comparing these age categorisations with the polynomial function of age as a continuous variable. (TIFF) [file pntd.0005265.s003.tiff]

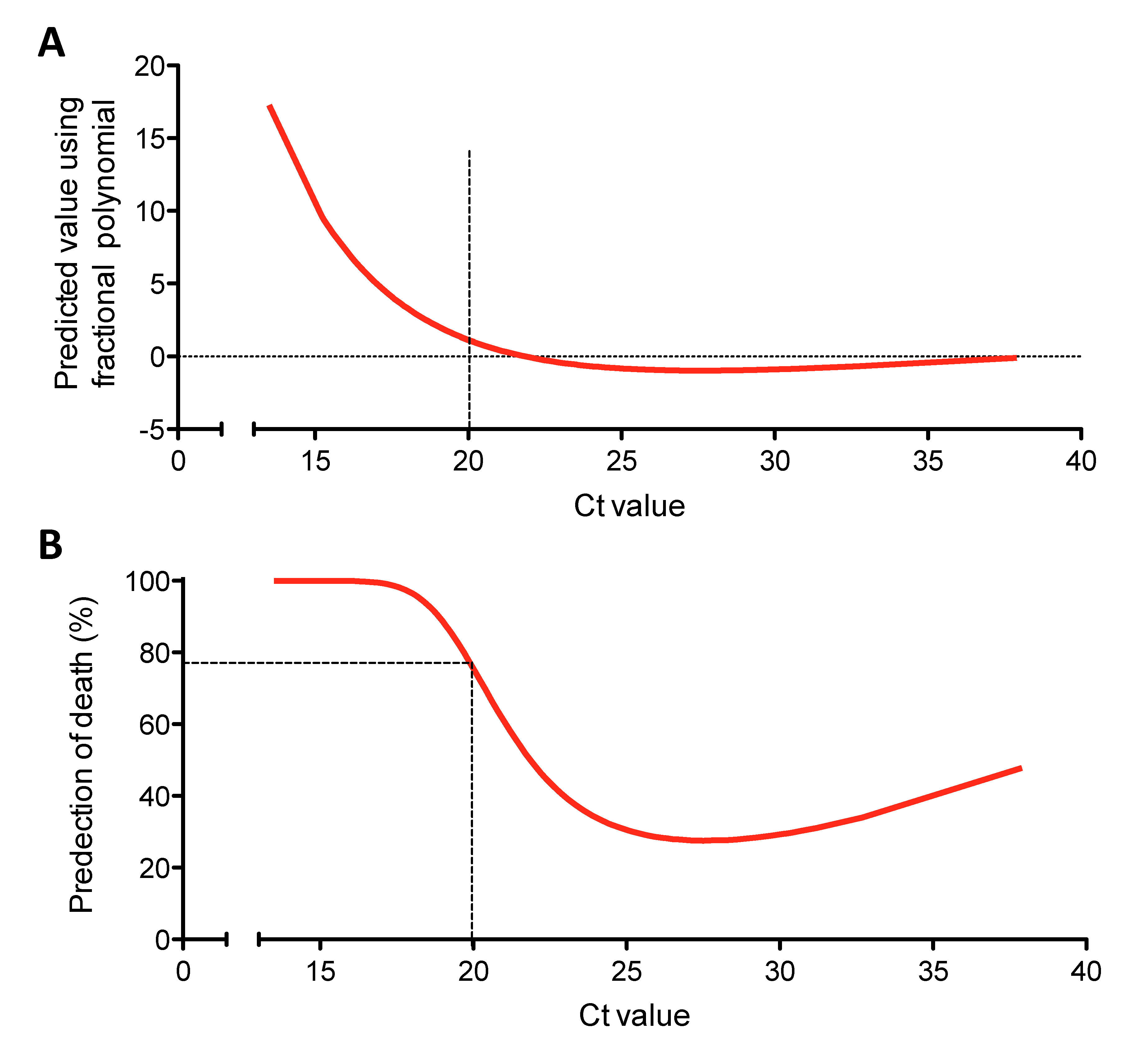

Supplement: S3 Fig — (A) Predicted risk of death using fractional polynomial analysis. (B) Predicted risk of death (%). The dotted vertical lines indicate the threshold for “high viral load” (Ct<20) and “low viral load” (Ct>20). (TIFF) [file pntd.0005265.s004.tiff]

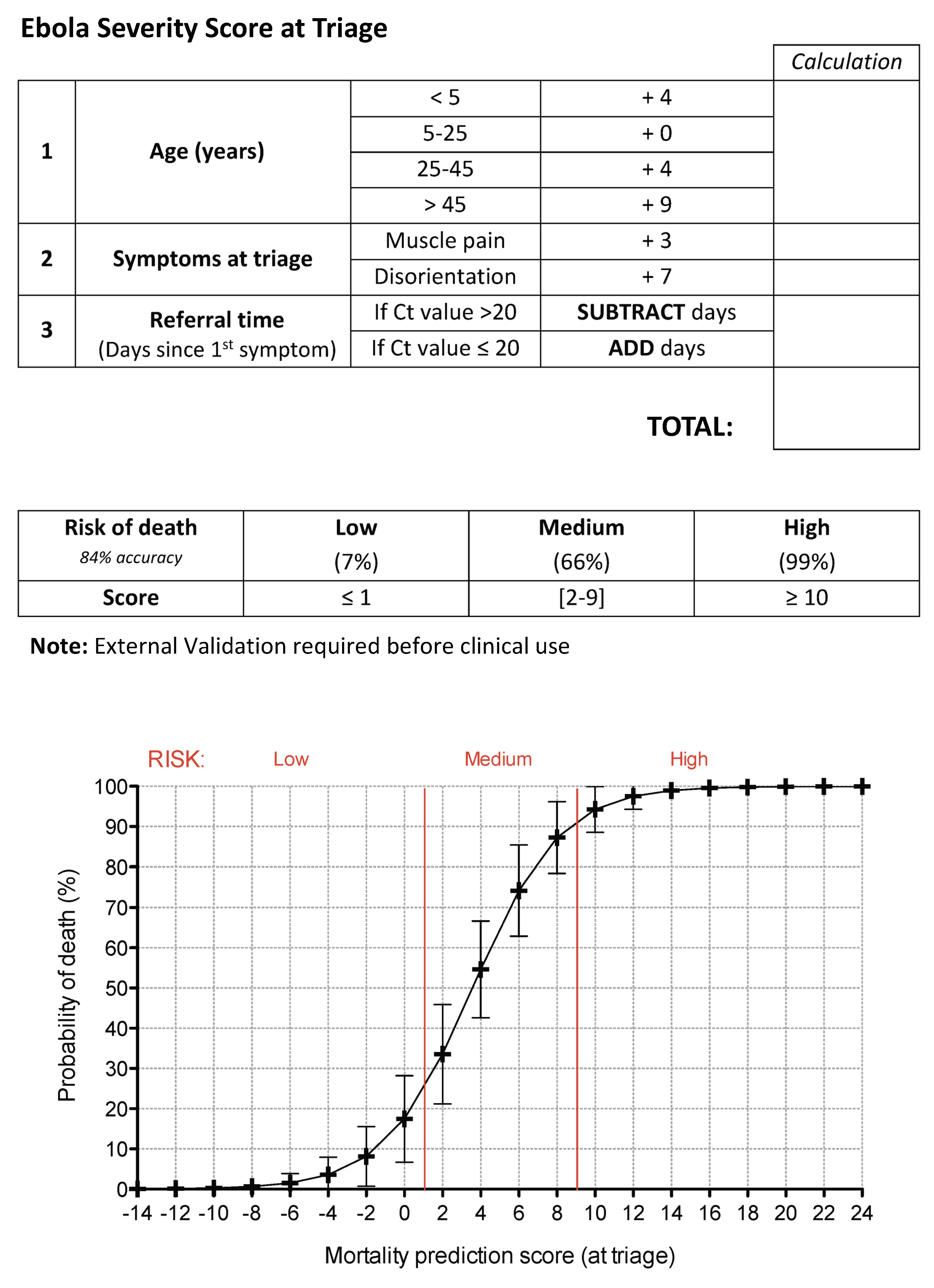

Supplement: S4 Fig — (TIFF) [file pntd.0005265.s005.tiff]

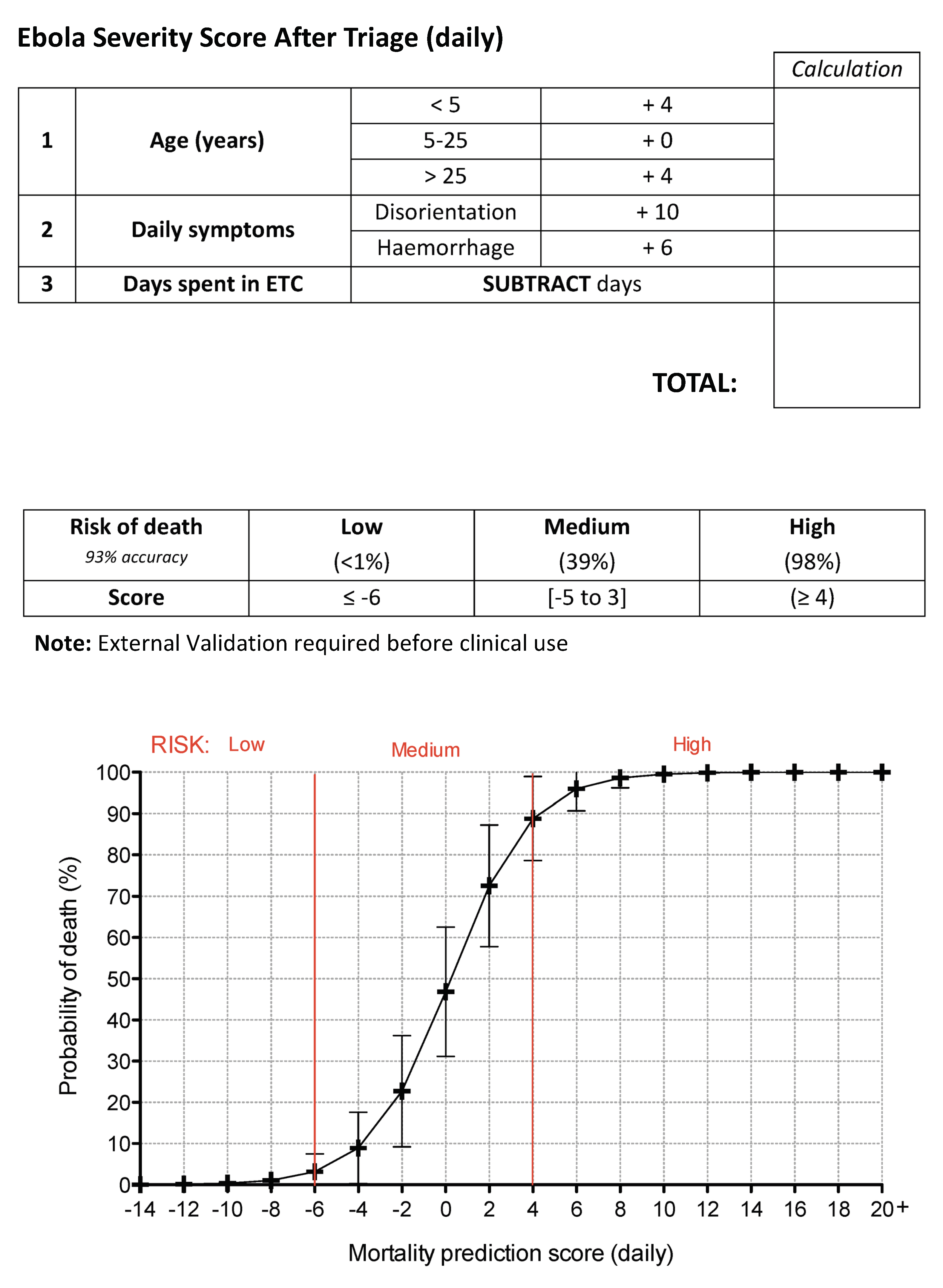

Supplement: S5 Fig — (TIFF) [file pntd.0005265.s006.tiff]
