# Supplementary material for: Predicting Ebola Severity: A Clinical Prioritization Score for Ebola Virus Disease
Source: PLoS Negl Trop Dis. 2017 Feb 2;11(2):e0005265. doi: 10.1371/journal.pntd.0005265 (PMC5289426; doi:10.1371/journal.pntd.0005265)
Supplement: S1 Table — (DOCX) [file pntd.0005265.s007.docx]

**Table S1**

| **Multivariate predictors for the outcome of death AT TRIAGE** | **OR** | **Coefficient** | **Std Error** | **p value** | **CI95%** | |
| --- | --- | --- | --- | --- | --- | --- |
| **Age (<5 and 25-45)** | 8.2 | 2.1 | 5.3 | 0.001 | 2.3 | 29.0 |
| **Age (>45)** | 80.9 | 4.4 | 87.0 | 0.000 | 9.8 | 665.4 |
| **Ct value (<20)** | 0.9 | -0.1 | 0.9 | 0.902 | 0.1 | 7.1 |
| **Referral time** | 0.6 | -0.5 | 0.1 | 0.000 | 0.4 | 0.8 |
| **Ct value (<20) X referral time** | 2.3 | 0.8 | 0.7 | 0.005 | 1.3 | 4.0 |
| **Myalgia** | 3.7 | 1.3 | 2.1 | 0.020 | 1.2 | 11.0 |
| **Disorientation** | 38.2 | 3.6 | 77.9 | 0.074 | 0.7 | 2077.5 |
| ***Intercept constant*** | *0.3* | *-1.3* | *0.2* | *0.095* | *0.1* | *1.3* |
