# Supplementary material for: Predicting Ebola Severity: A Clinical Prioritization Score for Ebola Virus Disease
Source: PLoS Negl Trop Dis. 2017 Feb 2;11(2):e0005265. doi: 10.1371/journal.pntd.0005265 (PMC5289426; doi:10.1371/journal.pntd.0005265)
Supplement: S2 Table — (DOCX) [file pntd.0005265.s008.docx]

**Table S2**

| **Multivariate predictors for the outcome of death AFTER TRIAGE** | **OR** | **Coefficient** | **Std Error** | **p value** | **CI95%** | |
| --- | --- | --- | --- | --- | --- | --- |
| **Age (<5 and >25)** | 8.1 | 2.1 | 6.8 | 0.012 | 1.6 | 42.1 |
| **Disorientation during admission** | 138.6 | 4.9 | 176.1 | 0.000 | 11.5 | 1671.7 |
| **Hemorrhage during admission** | 17.2 | 2.8 | 14.5 | 0.001 | 3.3 | 89.7 |
| **Days spent in ETC** | 0.5 | -0.6 | 0.1 | 0.000 | 0.4 | 0.7 |
| ***Intercept constant*** | 1.9 | 0.7 | 1.7 | 0.436 | 0.4 | 10.5 |
